# Differential Impacts of Prenatal Supplement Intake on Childhood Obesity Markers, Stratified by Gender and Other Prenatal Factors

**DOI:** 10.1155/jobe/3257488

**Published:** 2025-02-10

**Authors:** M. Batra, Y. Bekele, A. Halilagic, Y. Manios, G. Moschonis, B. Erbas

**Affiliations:** ^1^Department of Public Health, School of Psychology and Public Health, La Trobe University, Melbourne 3086, Victoria, Australia; ^2^Department of Reproductive Health, School of Public Health, Bahir Dar University, Bahir Dar 79, Ethiopia; ^3^Department of Paediatrics, Murdoch Children's Research Institute, Parkville 3052, Victoria, Australia; ^4^Department of Nutrition and Food Services, The Royal Children's Hospital, Parkville 3052, Victoria, Australia; ^5^Department of Food, Nutrition and Dietetics, School of Allied Health, Human Services and Sport, La Trobe University, Bundoora 3086, Victoria, Australia; ^6^Department of Nutrition and Dietetics, Hellenic Mediterranean University, Sitia 723008, Greece; ^7^Institute of Agri-Food and Life Sciences, Hellenic Mediterranean University Research Centre, Crete, Greece; ^8^La Trobe Institute for Sustainable Agriculture & Food (LISAF), La Trobe University, Melbourne, Australia

**Keywords:** childhood obesity, iron supplementation, perinatal epidemiology, prenatal micronutrient

## Abstract

**Objective:** To assess the association between maternal iron, folic acid and combined iron–folic acid (IFA) oral supplementation during pregnancy and childhood obesity markers in 9- to 13-year-olds.

**Methods:** Data from the 2007–2009 Healthy Growth Study were analysed. The study assessed obesity markers, i.e., body mass index (BMI), skinfold thickness and waist circumference. The research question was examined using generalised linear models stratified by the child's sex, maternal prepregnancy weight and gestational age.

**Results:** Folic acid and IFA supplements, but not iron alone, were significantly associated with lower waist circumference in all children (coef. −1.35, 95% CI: −2.47 to −0.23; coef. −1.01, 95% CI: −2.21 to −0.23, *p* < 0.05). These associations were observed only in girls with lower BMI (coef. −0.88), skinfold thickness (coef. −4.92) and waist circumference (coef. −2.99) with folic acid and similar IFA effects. Interestingly, in boys born to obese mothers before pregnancy, a significant negative association was observed for folic acid alone with BMI (coef. −3.55) and waist circumference (coef. −7.09) and IFA for the sum of skinfold thickness (coef. −19.68).

**Conclusion:** Maternal folic acid and IFA supplementation may contribute to a lower likelihood of childhood obesity, especially in girls and children of underweight or obese mothers, emphasising the importance of proper prenatal nutrition.

**Summary**

  What is already known?
• Oral folic and iron–folic acid intake during pregnancy could protect against childhood obesity, with some evidence suggesting sex-specific effects, but data on adolescents are limited.
  What does this study add?
• This study reveals that maternal folic acid alone and combined iron–folic acid (IFA) supplementation could reduce the odds of increased waist circumference in children, with significant effects primarily observed in girls. The study also highlights the differential impacts based on maternal prepregnancy weight and gestational age.
  How might these results change the direction of research or the focus of clinical practice?
• The findings highlight the importance of considering sex-specific effects and maternal factors like prepregnancy weight when assessing the role of prenatal supplementation on childhood obesity.
• Future research should explore synergies between mothers' behaviours during pregnancy and childhood physical activity that could potentially be used to inform more tailored and effective intervention strategies.

## 1. Introduction

Childhood obesity has become a global health crisis, with prevalence rising from 8% in 1990 to 20% in 2022, affecting over 390 million children and adolescents worldwide [1]. Prevalence is notably high in Mediterranean countries, including Greece, where 40% of children aged 6–9 years are overweight and 19%–24% are obese [2]. Obesity is linked to chronic diseases such as type 2 diabetes, cardiovascular diseases and cancers, imposing significant health and economic burdens [3, 4]. Without intervention, annual costs are projected to exceed $18 trillion by 2060 [5]. Understanding the determinants of childhood obesity is crucial for developing early effective prevention strategies.

Childhood obesity results from a complex interplay of genetic, environmental, behavioural and perinatal factors influencing energy balance and adiposity. Genetic predispositions play a significant role, with numerous gene variants linked to weight gain and adiposity [6]. Perinatal factors and early life experiences, such as maternal nutrition, gestational weight gain, birth outcomes, breastfeeding and infant feeding practices, are also associated with childhood obesity risk [7, 8]. Sociodemographic factors, including socioeconomic status, sex of the child, cultural norms and physical activity levels, further impact obesity risk [9, 10]. Understanding these synergistic effects is essential for resolving the complexities of childhood obesity.

The prenatal period is crucial for obesity and metabolic outcomes, with maternal nutrition quality and quantity significantly influencing birthweight and later obesity risk [11–13]. Micronutrient supplementation during pregnancy, including folate, iron, calcium and vitamins, has been linked to lower body fat, body mass index (BMI), skinfold thickness and waist circumference (WC) in children [14]. A study in the Netherlands showed that low maternal folate levels are associated with higher BMI in children aged 5–6 years [15]. Similarly, a study by Yajnik et al. [16] in India found an imbalance of low vitamin B12 and high folate levels at 28 weeks of gestation may increase adiposity in 6-year-old children [16].

The impact of specific micronutrient supplementation on childhood body composition beyond age 6 has been investigated, with studies yielding mixed results. A study in Nepal (*n* = 3771) reported that maternal supplementation with folic acid, iron and zinc reduced mean triceps skinfold thickness, subscapular skinfold thickness and arm fat area in children aged 6–8 years but did not show significant differences in groups' mean weight, BMI-for-age *z*-scores, WC or arm muscle area [17]. In contrast, a UK cohort study found no association between folate supplements administered between 18 and 32 weeks of gestation or folate taken at 32 weeks and the total body mass, fat mass or lean mass at 9 years of age, possibly due to residual confounding [18]. A recent meta-analysis of 8 studies (2022) suggests a small, nonsignificant effect of maternal folate-only intake on childhood obesity aged 7–15 years (effect size: 0.09, 95% CI:  = −0.03, 0.12) [19]. These inconsistent findings may arise from several methodological limitations, including reliance on serum folate levels, which are sensitive to recent intake, and insufficient adjustment for key confounders such as prepregnancy weight and smoking. Additionally, heterogeneity in study designs (e.g., cohort studies and randomised controlled trials), varying sample sizes (ranging from 63 to 5783 participants), and geographic differences in diet and sex distribution further contribute to these discrepancies [18, 20]. Furthermore, while iron and folic acid are often studied independently, combined iron–folic acid (IFA) supplementation is widely recommended in global maternal health programmes due to its potential synergistic effects on foetal growth and metabolism [21]. Evidence suggests that IFA may influence foetal growth patterns and adiposity regulation, indirectly contributing to reduced risks of childhood obesity [22, 23]. However, evidence remains limited and inconsistent, highlighting the need for further research to clarify these associations.

Sex-specific analysis is crucial, as boys and girls differ in fat distribution and metabolic responses to micronutrient supplementation. A Chinese study of 8016 mother–child pairs found an inverse relationship between BMI and skinfold thickness among girls aged 3–6.5 years whose mothers took multivitamins, iron and folic acid during pregnancy [24]. Both prepregnancy weight and weight gain during pregnancy are crucial factors. Maternal obesity can alter the intrauterine environment, impacting foetal development and increasing the offspring's risk of obesity [25, 26]. Obese women often experience relative folate deficiency due to chronic low-grade inflammation, which raises their metabolic need for folate (500 mcg/day to 5 mg/day for women with a BMI exceeding 30 kg/m^2^) [27, 28]. Therefore, maternal obesity may modify the relationship between prenatal micronutrient supplementation and childhood obesity, and it is essential to consider this factor when examining these associations.

Gestational age may also be important because preterm and full-term births have different developmental trajectories that can influence obesity risk [29]. This study investigates the relationship between prenatal micronutrient supplementation (iron, folic acid and IFA) and obesity markers (BMI, WC and skinfold thickness) in children aged 9–13 years. This age range was chosen because it represents a critical developmental window with relative stability in growth patterns, minimising confounding from pubertal changes. Additionally, the study explores potential differences based on biological sex, maternal prepregnancy weight and gestational age. A comprehensive assessment of these factors will provide insights into critical intervention periods and inform targeted prevention strategies.

## 2. Methods

This study utilised data from the Healthy Growth Study, a cross-sectional epidemiological study conducted between 2007 and 2009. The original study received ethical approval from the Ethics Committee of Harokopio University of Athens (approval no: 16/19.12.2006). The approval received also endorsed the prospective use of the study's deidentified data in future studies and secondary data analyses. All procedures adhered to the ethical principles outlined in the 1975 Declaration of Helsinki.

The study focused on students in the fifth and sixth grades of primary schools located within the municipalities in four counties covering the northern (i.e., Thessaloniki), central (i.e., Attica), western (i.e., Aitoloakarnania) and southern (i.e., Iraklio-Crete) parts of Greece. A multistage stratified random sampling approach was undertaken to achieve a representative sample. This method randomly selected municipalities and schools, stratifying them based on parental educational level and the total student population [30]. This ensured the inclusion of children from diverse backgrounds within the four countries. Following this process and after receiving approval from the Greek Ministry of Education, 77 primary schools were chosen to represent the broader area. Parents or guardians of eligible students were then contacted via letter to obtain their consent for participation. The overall response rate was 64.1%, resulting in a sample size of 2577 children aged 9–13 years. The details of the methodology are described elsewhere [31].

### 2.1. Sociodemographic and Perinatal Data

Researchers collected sociodemographic and perinatal data through interviews with parents during school visits. Standardised questionnaires minimised interviewer bias and ensured consistent data collection [30]. Phone interviews were used for a small portion (5%) of the sample and for those parents who could not attend the school visits. Parents also brought their children's birth certificates and medical records, which validated birth date, weight, gestational age, weight gain during infancy, delivery type and feeding patterns. Data on iron and folic acid intake during pregnancy (independent of each other) were obtained via a validated and reliable parental questionnaire. Data were separated into the first, second and third trimesters. Folate supplementation before pregnancy (i.e., the number of months prior) was also recorded.

### 2.2. Outcome Measures

Two trained members of the research teams conducted physical examinations and anthropometric measurements, including weight, height, BMI and WC, for each student. These measurements adhered to standardised procedures and used the same instruments across all schools to ensure consistency and reliability. These outcome measures were added as continuous variables in the analysis.

### 2.3. Statistical Analysis

The analysis investigated the relationship between binary exposure variables [iron intake (Y/N), folic acid intake (Y/N) or intake of both (Y/N) throughout pregnancy] and continuous outcome measures of infant body composition (BMI, WC and sum of skinfold thickness). Given the specific focus on children aged 9–13 years, a relatively homogeneous group, raw BMI scores were used as one of the outcome variables. This age range reduces variability in physical development, making standardisation less critical. While standardisation can be beneficial in some cases, it was not considered necessary for this study [32]. Initial model comparisons between standardised BMI *z*-scores and raw BMI data did not reveal significant differences in the results. Therefore, raw BMI scores were retained for the primary analysis, with standardised BMI *z*-scores included in sensitivity analyses to validate the robustness of the findings (Table S1).

Generalised linear models (GLMs) with a specific family (inverse Gaussian) and link function (identity) were chosen due to the skewed distribution of the body composition data (BMI, WC and sum of skinfold thickness). GLMs extend traditional linear regression models by allowing the response variable to follow distributions other than the normal distribution and by incorporating a link function to relate the mean of the response variable to the predictors. While standard linear regression assumes normally distributed residuals and constant variance (homoscedasticity), our diagnostic checks revealed significant positive skewness and heteroscedasticity in the data, making the assumptions of linear regression inappropriate.

The inverse Gaussian family was specifically selected after conducting comparative assessments of model fit using the Akaike information criterion (AIC) and Bayesian information criterion (BIC). These metrics are widely used for model selection and comparison because they balance model fit with model complexity, penalising overfitting caused by unnecessary parameters [33]. AIC prioritises identifying a model that fits the data well while controlling for complexity, whereas BIC applies a stronger penalty for additional parameters, making it particularly suitable for large sample sizes. Together, they provide complementary insights into model performance, ensuring that the selected model achieves an optimal balance between fit and simplicity. The identity link function was chosen to maintain the interpretability of the coefficients in their original scale, ensuring clarity in the presentation of results.

Covariates were included in the model based on a directed acyclic graph (DAG) to account for other factors influencing the results (Figure S1). These covariates included prepregnancy weight, gestational diabetes, hypertension, breastfeeding duration, smoking habits, maternal age, education, pregnancy weight gain and gestational age. Notably, the method of conception was automatically excluded by the model fitting process to avoid issues with interrelated variables (multicollinearity). Robust standard errors were added to account for unequal variances, and bootstrapping was used to assess the model's reliability. Results were reported using coefficients and 95% confidence intervals (CIs) presented for each exposure variable. The coefficient quantifies the expected change in the mean of the outcome variable when the exposure changes from the reference category (no) to the nonreference category (yes) while adjusting for other covariates in the model. Further, stratification was done by biological sex, mother's prepregnancy weight and gestational age.

BMI was further categorised into normal weight, overweight and obesity groups using the WHO-defined BMI *z*-score cut-offs: normal weight (*z*-score between −2 SD and +1 SD), overweight (*z*-score > +1 SD and ≤ +2 SD) and obesity (*z*-score > +2 SD) [34]. Multinomial logistic regression models were applied to explore associations between binary maternal supplementation variables (iron, folic acid and IFA) and these categorical BMI outcomes, stratified by biological sex (boys and girls).

The results of this analysis are presented in a forest plot, summarising relative risk ratios (RRR) and 95% CIs across subgroups. The subgroup analysis allows the visualisation of gender-specific patterns and highlights differences in supplementation effects across BMI categories. These findings complement the continuous outcome analysis and offer additional insights into the complex relationships between maternal supplementation and child anthropometric outcomes. All analyses were performed using STATA software at a significance level of *p* < 0.05.

## 3. Results

Among those taking the iron-alone supplement, 42% (*n* = 266) had education exceeding 14 years, followed by 9–14 years (41.1%, *n* = 260) and less than 9 years (16.9, *n* = 107). Similar patterns were observed in folic acid and IFA groups. Regarding prepregnancy weight status, the majority of participants in the iron-alone group were normal weight (76.9%, *n* = 492), followed by overweight (*n* = 84, 13.1%), underweight (*n* = 42, 6.6%) and obese (*n* = 22, 3.4%), comparable distributions were found in folic acid-alone and IFA groups. More than three-fourths of participants in the iron-alone group (*n* = 524, 81.9%), folic acid-alone group (*n* = 217, 81.6%) and IFA group (*n* = 184, 83.6%) did not smoke during pregnancy. In terms of parity, nearly half of the participants in the iron-alone group (*n* = 326, 50.9%), the folic acid-alone group (*n* = 133, 50.0%) and the IFA group (*n* = 104, 47.3%) were multiparous.

Regarding gestational diabetes, the majority of participants in the iron-alone group (*n* = 608, 95.2%), the folic acid-alone group (*n* = 254, 95.5%) and the IFA group (*n* = 208, 94.6%) did not develop gestational diabetes. However, a small proportion experienced gestational diabetes: 3.3% (*n* = 21) in the iron-alone group, 3.0% (*n* = 8) in the folic acid-alone group and 3.6% (*n* = 8) in the IFA group.

In terms of income distribution, the majority of participants in the iron-alone group earned between 12,000€ and 30,000€ (*n* = 325, 55.5%), followed by those earning greater than 30,000€ (*n* = 165, 28.2%) and less than 12,000 (*n* = 96, 16.4%). Similar patterns were observed in folic acid-alone and IFA groups.

As for biological sex, 53.1% (*n* = 340) of those in the iron-alone group were male, along with 53.4% (*n* = 142) folic acid-alone group and 55% (*n* = 121) in the IFA group. The majority of the participants in iron-alone group were appropriate weight for gestational age (*n* = 516, 80.6%), followed by small for gestational age (SGA) (*n* = 77, 12%) and large for gestational age (*n* = 47, 7.3%). Similar trends were observed in the folic acid-alone and IFA groups.

Regarding BMI categories of offspring, most children were classified as having normal weight (47.7%, *n* = 1259). Overweight was observed in 29.3% (*n* = 773) of children overall, with similar distributions across groups: 29.1% (*n* = 185) in the iron-alone group, 29.6% (*n* = 78) in the folic acid-alone group and 30.1% (*n* = 66) in the IFA group. Obesity was noted in 22.1% (*n* = 582) of children overall, with 20.9% (*n* = 133) in the iron-alone group, 18.6% (*n* = 49) in the folic acid-alone group and 20.1% (*n* = 44) in the IFA group (Table 1).

For iron intake, BMI was identical for both intake (*n* = 640, 20.3 ± 3.9) and nonintake (*n* = 1695, 20.3 ± 3.8) groups. The sum of skinfold thickness (53.8 ± 22.3 vs. 55.5 ± 23.1) and WC (68.61 ± 9.70 vs. 69.45 ± 10.17) were both slightly lower in the intake group compared to the nonintake group. For folic acid intake, BMI (19.9 ± 3.6 vs. 20.3 ± 3.9), the sum of skinfold thickness (51.8 ± 20.9 vs. 55.5 ± 23.1) and WC (67.53 ± 8.98 vs. 69.00 ± 9.75) were all lower in the intake group (*n* = 266) compared to the nonintake group (*n* = 2069). For combined iron and folic acid intake, the intake group (*n* = 220) had a slightly lower BMI (20.1 ± 3.7 vs. 20.3 ± 3.8), the sum of skinfold thickness (52.6 ± 21.6 vs. 55.3 ± 23.0) and WC (67.89 ± 9.13 vs. 68.93 ± 9.72) compared to the nonintake group (*n* = 2115) (Table 2).

In regression analysis, iron alone, folic acid alone and combined IFA intake during pregnancy were not significantly associated with childhood BMI or skinfold thickness in the overall sample. However, folic acid alone and IFA intake were significantly associated with reduced WC in all children (folic acid alone: coefficient −1.35, 95% CI: −2.47 to −0.23, *p*=0.018; IFA: coefficient −1.01, 95% CI: −2.21 to −0.23, *p*=0.028).

Stratified by biological sex, significant negative associations in BMI were found only among girls for folic acid alone (coefficient −0.88, 95% CI: −1.45 to −0.29, *p*=0.003) and IFA intake (coefficient −0.77, 95% CI: −1.42 to −0.12, *p*=0.020). For skinfold thickness, significant reductions were observed only among girls for iron alone (coefficient −3.29, 95% CI: −5.79 to −0.78, *p*=0.010), folic acid alone (coefficient −4.92, 95% CI: −8.25 to −1.60, *p*=0.004) and IFA intake (coefficient −4.82, 95% CI: −8.53 to −1.10, *p*=0.011). Similarly, WC reductions were evident only among girls (iron alone: coefficient −1.01, 95% CI: −2.21 to −0.00, *p*=0.049; folic acid alone: coefficient −2.99, 95% CI: −4.34 to −1.65, *p* < 0.001; IFA: coefficient −2.93, 95% CI: −4.40 to −1.46, *p* < 0.001), with no significant effects observed for boys (Table 3).

**Figure 1:**
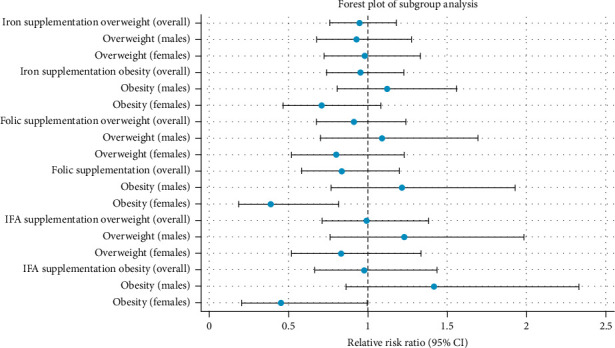
Associations between maternal supplement intake and childhood BMI categories; overall and sex-specific analysis.

The forest plot (Figure 1) depicts subgroup associations, presenting RRR for overweight and obesity categories, with the normal weight group as the reference. Folic acid intake was significantly associated with reduced odds of obesity among females (RRR: 0.39, 95% CI: 0.18 to 0.81, *p*=0.012). Similarly, IFA intake was linked to lower odds of obesity (RRR: 0.45, 95% CI: 0.20 to 0.99, *p*=0.049).

Table 4 presents the relationship between pregnancy supplement intake (iron alone, folic acid alone and IFA) and childhood obesity outcomes across different maternal prepregnancy weight categories (normal weight as the reference category, underweight, overweight and obese). Iron supplementation alone showed no significant association with childhood obesity measures across maternal prepregnancy weight categories. Folic acid intake displayed variable associations: children of underweight mothers had lower BMI (coefficient: −1.35, 95% CI: −2.61 to −0.08, *p*=0.036), skinfold thickness (coefficient: −8.92, 95% CI: −15.27 to −2.57, *p*=0.006) and WC (coefficient: −3.43, 95% CI: −6.15 to −0.71, *p*=0.013). Among children of mothers with obesity, folic acid intake was associated with lower BMI (coefficient: −4.17, 95% CI: −6.34 to −1.99, *p* < 0.001) and WC (coefficient: −10.64, 95% CI: −15.66 to −5.61, *p* < 0.001). Similarly, IFA intake during pregnancy significantly reduced BMI (coefficient: −3.51, 95% CI: −5.63 to −1.39, *p*=0.001) and WC (coefficient: −10.29, 95% CI: −15.88 to −4.72, *p* < 0.001) in children of mothers with obesity before pregnancy.

When stratified by gender and maternal prepregnancy weight (Table 5), significant associations were observed. Among girls born to underweight mothers, folic acid alone and IFA intake were associated with reduced WC (folic acid alone: coefficient −3.44, 95% CI: −6.51 to −0.37, *p*=0.028; IFA: coefficient −4.07, 95% CI: −7.44 to −0.69, *p*=0.018). In contrast, among boys born to mothers with obesity before pregnancy, folic acid alone was significantly associated with lower BMI (coefficient −3.55, 95% CI: −6.25 to −0.85, *p*=0.010) and reduced WC (coefficient −7.09, 95% CI: −14.03 to −0.15, *p*=0.045). Additionally, IFA intake was associated with a significant reduction in the sum of skinfold thickness (coefficient −19.68, 95% CI: −36.19 to −3.16, *p*=0.020).

When stratified by gender and gestational age (Table 6), significant associations were observed among girls born SGA. Maternal iron intake alone was associated with lower BMI in these girls (coefficient −1.67, 95% CI: −3.22 to −0.11, *p*=0.036). Folic acid alone and IFA intake were significantly negatively associated with BMI (folic acid alone: coefficient −3.11, 95% CI: −4.46 to −1.77, *p* < 0.001; IFA: coefficient −3.47, 95% CI: −4.76 to −2.17, *p* < 0.001), the sum of skinfold thickness (folic acid alone: coefficient −20.22, 95% CI: −26.25 to −14.19, *p* < 0.001; IFA: coefficient −20.55, 95% CI: −26.46 to −14.64, *p* < 0.001), and WC (folic acid alone: coefficient −8.28, 95% CI: −11.50 to −5.05, *p* < 0.001; IFA: coefficient −8.31, 95% CI: −11.63 to −4.99, *p* < 0.001) exclusively among girls born SGA.

## 4. Discussion

This study examined the influence of iron and folic acid supplementation (alone or combined) during pregnancy on childhood obesity markers (BMI, skinfold thickness and WC). While overall associations with BMI and skinfold thickness were absent, folic acid alone and IFA supplementation were linked to a smaller WC in all children. Interestingly, these effects were primarily present in girls, where folic acid alone and IFA intake were associated with lower levels of BMI and skinfold thickness. Additionally, maternal supplementation's impact varied across all categories of maternal prepregnancy weight and among infants born SGA.

This is the first study to examine the relationship between maternal supplementation and childhood obesity in children older than 9 years. Our study extends previous research [17, 35] by revealing a possible link between folic acid supplementation (alone or combined with iron) and reduced WC in all children. This association with WC might be explained by folic acid's role in cellular growth, DNA methylation and one-carbon metabolism [36, 37]. Folic acid acts as a coenzyme, influencing the balance between molecules regulating gene expression and body fat mass. Further research is needed to explore how folic acid supplementation during pregnancy influences childhood body composition, particularly WC, even if BMI and skinfold thickness are not significantly affected.

Our findings suggest that the effects of maternal supplementation on childhood obesity might be sex-specific, with potential implications for girls. While research on the long-term effects of maternal supplementation on offspring health is limited, particularly in this age group (9–13 years), emerging evidence points to sex-specific influences. For example, animal studies have shown that maternal folic acid can impact body weight and insulin sensitivity differently in female offspring [38]. Additionally, hormonal factors, such as leptin, which plays a role in appetite regulation, exhibit sex differences and may interact with folic acid metabolism [39]. In our study, the observed association with WC was primarily evident in girls. This may be influenced by a combination of genetic and environmental factors. Although we observed no significant association with obesity measures in boys, factors like sociocultural influences on diet and body image might contribute to these gender differences. Future research should delve deeper into sex-specific effects and consider potential modifying factors like childhood dietary patterns and physical activity levels.

Stratifying the analysis by prepregnancy weight revealed intriguing interactions between maternal weight status and the effects of maternal supplementation on childhood obesity markers. Folic acid supplementation (alone or combined with iron) was inversely associated with different obesity measures depending on the mother's weight before pregnancy. Children born to obese mothers who received folic acid supplementation exhibited a lower BMI compared to those who did not. This might be due, in part, to the known association between prepregnancy obesity and folate deficiencies [40]. Folate plays a crucial role in numerous biological processes, and adequate levels during pregnancy could influence offspring body composition, particularly in maternal obesity. For offspring of underweight mothers, folic acid supplementation was associated with a reduced sum of skinfold thickness. Studies suggest that prepregnancy underweight can impact placental gene expression, potentially affecting the development of obesity in children [41]. Folic acid supplementation during pregnancy might play a role in mitigating these potential effects for underweight mothers. Interestingly, for both underweight and obese mothers, folic acid supplementation was associated with a lower WC in their children. This finding suggests a broader potential benefit of folic acid on body fat distribution, regardless of maternal weight category. Such findings align with the established role of folic acid in DNA synthesis and repair, essential processes during foetal development that may influence long-term metabolic health [42]. In contrast, iron alone did not show a significant association with childhood obesity across any prepregnancy weight category. This could be due to pre-existing iron depletion in mothers, commonly caused by menstruation and inadequate dietary iron intake. Furthermore, increased iron demands during pregnancy further exacerbate this depletion [43]. These combined factors could mask iron supplementation's independent effects on childhood obesity.

Interestingly, the associations with folic acid supplementation were not observed in our sample of overweight women. One possible explanation is that obese women tend to have higher metabolic needs and a greater likelihood of folate deficiencies, which might make them more responsive to supplementation. Previous studies have shown that while the benefits of folic acid supplementation are clear in obese women, they are less consistent in overweight women [27].

Stratification analysis by girls and prepregnancy weight gain revealed that supplementation with iron alone and folic acid alone is negatively associated with all obesity categories in children born to mothers with normal prepregnancy weight. Conversely, in boys born to mothers who were obese before pregnancy, folic acid alone was significantly associated with reduced BMI and WC, while the IFA was linked to a substantial decrease in the sum of skinfold thickness. This trend may be affected by genetics, maternal nutrition status and childhood feeding practices. Research indicates that micronutrient deficiency before pregnancy, as well as maternal iron status during pregnancy, is linked with cardiometabolic disorders among females [44]. These factors might contribute to the observed variations in how boys and girls respond to iron and folic acid supplementation. However, future longitudinal studies are needed to understand better these complex interactions and the differing effects on boys and girls.

Our findings align with a previous study in preschoolers, demonstrating that maternal folic acid supplementation during pregnancy protects against childhood obesity in girls born SGA but not boys [24]. This sex-specific effect highlights the importance of considering both gestational ages. This observed protection in SGA girls could be due to the critical role micronutrients like folic acid play in foetal development and growth. SGA infants are particularly susceptible to later metabolic complications, and adequate micronutrient intake may mitigate this risk. Future research should explore the long-term health outcomes of these children and delve deeper into the mechanisms behind these associations. Potential explanations for the protective effect might include improved prenatal outcomes, reduced micronutrient deficiencies, enhanced immune function, lower rates of anaemia and better early childhood nutrition [45–47]. Conversely, folic acid and IFA supplementation have been linked to reduced vulnerability to adverse pregnancy outcomes, micronutrient deficiencies and childhood infections [47, 48]. These factors all contribute significantly to the development of childhood obesity.

This study's strength lies in its extensively identified target groups, allowing for tailored interventions. The sample was predominantly comprised of Caucasian Greek children; this homogeneity offers advantages by controlling for numerous unmeasured confounding factors. Sensitivity analyses excluding the small non-Caucasian subgroup (< 2%) revealed minimal impact on the results, with less than 10% differences and therefore not reported. Additional strengths include a large and potentially representative sample, using health records to obtain perinatal data (reducing recall bias for some information), and using trained researchers with standardised procedures. However, limitations remain. Iron, folic acid and combined supplementation data rely on maternal recall, which can introduce bias. The cross-sectional study design precludes definitive causal inferences. Although multiple comparisons were conducted, the focus was on identifying potential trends and associations rather than establishing definitive causal relationships. Uniquely, this study investigates children over 9 years old, a group not previously examined in this context.

## 5. Conclusions

Our findings reveal a complex relationship between maternal folic acid and iron supplementation and childhood obesity markers, with notable variations based on biological sex and maternal prepregnancy weight. While these supplements are widely prescribed, their efficacy in preventing childhood obesity may be influenced by adherence rates, which remain largely unmonitored. To optimise potential benefits, public health interventions should prioritise improving women's understanding of the importance of these supplements in women's and their offspring' health alongside tailored nutritional guidance during pregnancy, especially among women with lower health literacy. Further research is necessary to elucidate the underlying mechanisms and long-term health implications.

## Figures and Tables

**Table 1:** Demographic, pregnancy and infant characteristics by maternal supplement intake.

Factors	Daily supplement intake during the entire pregnancy, *n* (%)		
	Iron alone, 640 (27.4)	Folic acid alone, 266 (11.4)	IFA, 220 (9.4)
Maternal			
Age, mean (SD) years	39.8 (4.8)	40.5 (4.6)	40.5 (4.7)
Educational level, *n* (%)			
< 9 years, 573 (22.2)	107 (16.9)	36 (13.6)	29 (13.2)
9–14 years, 1010 (39.2)	260 (41.1)	82 (30.9)	68 (31.1)
> 14 years, 994 (38.6)	266 (42.0)	147 (55.5)	122 (55.7)
Prepregnancy weight status, *n* (%)			
Normal weight, 1744 (74.7)	492 (76.9)	202 (75.9)	165 (75.0)
Underweight, 159 (6.8)	42 (6.6)	23 (8.7)	18 (8.2)
Overweight, 338 (14.5)	84 (13.1)	36 (13.5)	33 (15.0)
Obese, 94 (4.0)	22 (3.4)	5 (1.9)	4 (1.8)
Weight gain during pregnancy kgs, mean (SD)	14.8 (8.9)	14.6 (8.9)	14.6 (9.1)
Smoking during pregnancy, *n* (%)			
Yes, 373 (15.9%)	116 (18.1)	49 (18.4)	36 (16.4)
No, 1969 (84.1%)	524 (81.9)	217 (81.6)	184 (83.6)
Parity, *n* (%)			
Uniparous, 1440 (55.1)	314 (49.1)	133 (50.0)	116 (52.7)
Multiparous, 1773 (44.9)	326 (50.9)	133 (50.0)	104 (47.3)
Gestational diabetes, *n* (%)			
Yes, 58 (2.5)	21 (3.3)	8 (3.0)	8 (3.6)
No, 2206 (95.0)	608 (95.2)	254 (95.5)	208 (94.6)
Pregnancy hypertension, *n* (%)			
Yes, 74 (3.2)	25 (3.9)	13 (4.9)	13 (5.9)
No, 2195 (94.3)	604 (94.5)	252 (94.7)	206 (93.6)
Familial			
Income, *n* (%)			
< 12,000€, 481 (22.7)	96 (16.4)	29 (11.8)	26 (12.9)
12,000–30000€, 1070 (50.5)	325 (55.5)	134 (54.7)	108 (53.5)
> 30,000€, 569 (26.8)	165 (28.2)	82 (33.5)	68 (33.7)
Residence, *n* (%)			
Metropolitan, 1729 (64.7)	409 (63.9)	180 (67.7)	148 (67.3)
Semiurban, 453 (16.9)	110 (17.2)	46 (17.3)	40 (18.2)
Rural, 492 (18.4)	121 (18.9)	40 (15.0)	32 (14.6)
Offspring			
Biological sex, *n* (%)			
Boy, 1348 (50.6)	340 (53.1)	142 (53.4)	121 (55.0)
Girl, 1318 (49.4)	300 (46.9)	124 (46.6)	99 (45.0)
Weight categories for gestational age, *n* (%)			
Appropriate, 1885 (80.5)	516 (80.6)	214 (80.5)	175 (79.6)
Small, 284 (12.1)	77 (12.0)	37 (13.9)	32 (14.6)
Large, 173 (7.4)	47 (7.3)	15 (5.6)	13 (5.9)
Exclusive breastfeeding duration, mean (SD) months	1.1 (1.7)	1.1 (1.6)	1.0 (1.6)
BMI categories, *n* (%)			
Underweight (< −2SD), 24 (0.9)	7 (1.1)	3 (1.1)	3 (1.4)
Normal weight, 1259 (47.7)	310 (48.8)	134 (50.8)	106 (48.4)
Overweight (> +1SD), 773 (29.3)	185 (29.1)	78 (29.6)	66 (30.1)
Obesity (> + 2SD), 582 (22.1)	133 (20.9)	49 (18.6)	44 (20.1)

*Note:* 58 (2.5%) and 53 (2.3%) participants responded “don't know” to gestational diabetes and pregnancy hypertension, respectively.

Abbreviations: HTN = hypertension, IFA = iron–folic acid, SD = standard deviation.

**Table 2:** Childhood obesity markers by maternal supplement intake.

Pregnancy supplement intake	Childhood outcomes, mean (SD)		
	BMI (kg/m^2^)	The sum of skinfold thickness (mm)	Waist circumference (cm)
Iron alone			
Yes, *n* = 640	20.3 (3.9)	53.8 (22.3)	68.61 (9.70)
No, *n* = 1695	20.3 (3.8)	55.5 (23.1)	69.45 (10.17)
Folic acid alone			
Yes, *n* = 266	19.9 (3.6)	51.8 (20.9)	67.53 (8.98)
No, *n* = 2069	20.3 (3.9)	55.5 (23.1)	69.00 (9.75)
IFA			
Yes, *n* = 220	20.1 (3.7)	52.6 (21.6)	67.89 (9.13)
No, *n* = 2115	20.3 (3.8)	55.3 (23.0)	68.93 (9.72)

Abbreviations: BMI = body mass index, cm = centimetre, IFA = iron–folic acid, kg = kilogram, m^2^ = metre square, mm = millimetre, SD = standard deviation.

**Table 3:** Impact of maternal supplement intake on childhood obesity markers: overall and sex-specific analysis.

Pregnancy supplement intake	Childhood outcomes, coefficient (95% CI), *p* value								
	BMI			The sum of skinfold thickness			Waist circumference		
	All *n* = 2241	Boys *n* = 1106	Girls *n* = 1135	All *n* = 2216	Boys *n* = 1094	Girls *n* = 1122	All *n* = 2216	Boys *n* = 1094	Girls *n* = 1122
Iron alone, yes	−0.14 (−0.48, 0.19), 0.412	0.09 (−0.41, 0.59), 0.725	−0.40 (−0.86, 0.05), 0.080	−1.44 (−3.53, 0.64), 0.176	0.06 (−3.15, 3.27), 0.968	**−3.29 (−5.79, −0.78), 0.010**⁣^∗^	0.49 (−1.33, 0.36), 0.260	0.18 (−1.01, 1.47), 0.783	**−1.01 (−2.21, −0.00), 0.049**⁣^∗^
Folic acid alone, yes	−0.29 (−0.75, 0.16), 0.209	0.25 (−0.43, 0.93), 0.476	**−0.88 (−1.45, −0.29), 0.003**⁣^∗^	−1.73 (−4.59, 1.14), 0.238	0.94 (−3.47, 5.63), 0.675	**−4.92 (−8.25, −1.60), 0.004**⁣^∗^	**−1.35 (−2.47, −0.23), 0.018**⁣^∗^	0.30 (−1.45, 2.06), 0.734	**−2.99 (−4.34, −1.65), < 0.001**⁣^∗^
IFA, yes	−0.10 (−0.60, 0.40), 0.695	0.48 (−0.26, 1.21), 0.202	**−0.77 (−1.42, −0.12), 0.020**⁣^∗^	−0.72 (−3.90, 2.46), 0.659	2.56 (−2.26, 7.38), 0.297	**−4.82 (−8.53, −1.10), 0.011**⁣^∗^	**−1.01 (−2.21, −0.23), 0.028**⁣^∗^	0.83 (−1.06, 2.71), 0.391	**−2.93 (−4.40, −1.46), < 0.001**⁣^∗^

*Note:* Bold at *p* value ≤ 0.05.

Abbreviations: BMI = body mass index, CI = confidence interval, IFA = iron–folic acid.

⁣^∗^Statistically significant.

**Table 4:** Impact of maternal supplement intake on childhood obesity markers: stratified by maternal prepregnancy weight.

Childhood outcome	Pregnancy supplement intake	Maternal prepregnancy weight coefficient (95% CI), *p* value			
		Normal weight (*n* = 1744)	Underweight (*n* = 159)	Overweight (*n* = 338)	Obese (*n* = 94)
BMI	Iron alone, *n* = 640	−0.13 (−0.50, 0.24), 0.494	−0.30 (−1.51, 0.90), 0.621	−0.50 (−1.58, 0.56), 0.358	0.61 (−1.71, 2.92), 0.608
	Folic acid alone, *n* = 266	−0.19 (−0.68, 0.30), 0.446	**−1.35 (−2.61, −0.08), 0.036**⁣^∗^	−0.78 (−0.72, 2.28), 0.307	**−4.17 (−6.34, −1.99), 0.001**⁣^∗^
	IFA, *n* = 220	0.01 (−0.53, 0.55), 0.966	−1.27 (−2.84, 0.29), 0.112	0.69 (−0.92, 2.31), 0.396	**−3.51 (−5.63, −1.39), 0.001**⁣^∗^

The sum of skinfold thickness	Iron alone, *n* = 640	−1.04 (−3.40, 1.32), 0.387	−4.99 (−14.03, 4.03), 0.278	−4.08 (−10.59, 2.41), 0.218	8.09 (−3.71, 19.9), 0.179
	Folic acid alone, *n* = 266	−1.20 (−4.47, 2.05), 0.469	**−8.92 (−15.27, −2.57), 0.006**⁣^∗^	3.27 (−6.33, 12.88), 0.504	−14.55 (−29.39, 0.29), 0.055
	IFA, *n* = 220	−0.21 (−3.83, 3.40), 0.909	**−9.15 (−18.02, −0.28), 0.043**⁣^∗^	3.49 (−6.77, 13.77), 0.504	−16.94 (−37.97, 4.09), 0.114

Waist circumference	Iron alone, *n* = 640	−0.53 (−1.45, 0.38), 0.254	−0.62 (−3.51, 2.28), 0.676	−1.27 (−3.97, 1.41), 0.354	2.66 (−3.22, 8.54), 0.376
	Folic acid alone, *n* = 266	−1.19 (−2.43, 0.05), 0.059	**−3.43 (−6.15, −0.71), 0.013**⁣^∗^	−1.28 (−2.33, 4.90), 0.486	**−10.64 (−15.66, −5.61), < 0.001**⁣^∗^
	IFA, *n* = 220	−0.84 (−2.17, 0.49), 0.215	−**3.67 (−6.96, −0.39), 0.029**⁣^∗^	1.51 (−2.36, 5.37), 0.445	**−10.29 (−15.88, −4.72), < 0.001**⁣^∗^

Abbreviations: BMI = body mass index, CI = confidence interval, IFA = iron–folic acid.

⁣^∗^Statistically significant results are bolded (*p* ≤ 0.05).

**Table 5:** Impact of maternal supplement intake on childhood obesity markers: stratified by gender and prepregnancy weight.

Pregnancy supplement intake	Stratification	BMI		The sum of skinfold thickness		Waist circumference	
	Gender and maternal prepregnancy weight	Coefficient (95% CI)	*p* value	Coefficient (95% CI)	*p* value	Coefficient (95% CI)	*p* value
Iron alone, yes	Boys (underweight), *n* = 83	−0.55 (−2.24, 1.14)	0.524	−0.14 (−11.20, 10.91)	0.980	−0.53 (−4.89, 3.82)	0.810
	Girls (underweight), *n* = 76	0.00 (−1.66, 1.66)	0.999	−6.55 (−14.71, 1.60)	0.115	−1.28 (−5.55, 2.99)	0.556
	Boys (overweight), *n* = 165	−0.70 (−2.14, 0.73)	0.336	−4.93 (−15.52, 5.66)	0.362	−0.65 (−4.16, 2.86)	0.717
	Girls (overweight), *n* = 179	−0.69 (−2.12, 0.74)	0.345	**−7.83 (−14.92, −0.73)**⁣^∗^	**0.031**⁣^∗^	−2.69 (−6.33, 0.956)	0.148
	Boys (obese), *n* = 47	1.29 (−1.95, 4.53)	0.435	7.32 (−13.47, 28.11)	0.490	1.76 (−6.39, 9.89)	0.672
	Girls (obese), *n* = 47	1.35 (−2.41, 5.10)	0.483	12.32 (−7.63, 32.26)	0.226	5.54 (−1.46, 12.55)	0.121

Folic acid alone, yes	Boys (underweight), *n* = 83	−1.82 (−4.10, 0.47)	0.120	−6.11 (−20.15, 7.92)	0.393	−3.79 (−8.74, 1.17)	0.134
	Girls (underweight), *n* = 76	−1.15 (−2.33, 0.02)	0.054	−7.56 (−16.27, 1.14)	0.089	**−3.44 (−6.51, −0.37)**⁣^∗^	**0.028**⁣^∗^
	Boys (overweight), *n* = 165	0.62 (−1.59, 2.83)	0.580	2.62 (−11.65, 16.89)	0.719	3.64 (−1.26, 8.54)	0.145
	Girls (overweight), *n* = 179	0.29 (−1.74, 2.33)	0.774	−3.15 (−14.76, 8.46)	0.595	−2.51 (−7.69, 2.67)	0.343
	Boys (obese), *n* = 47	**−3.55 (−6.25, −0.85)**⁣^∗^	**0.010**⁣^∗^	−14.05 (−28.44, 0.34)	0.056	**−7.09 (−14.03, −0.15)**⁣^∗^	**0.045**⁣^∗^
	Girls (obese), *n* = 47	−1.14 (−7.83, 5.55)	0.739	15.69 (−28.84, 60.21)	0.490	−3.76 (−19.17, 11.65)	0.632

IFA, yes	Boys (underweight), *n* = 83	−1.69 (−4.74, 1.35)	0.276	−7.68 (−22.45, 7.09)	0.308	−3.89 (−10.21, 2.42)	0.227
	Girls (underweight), *n* = 76	−1.09 (−2.54, 0.35)	0.139	−8.85 (−17.96, 0.25)	0.057	**−4.07 (−7.44, −0.69)**⁣^∗^	**0.018**⁣^∗^
	Boys (overweight), *n* = 165	0.75 (1.50, 3.00)	0.513	3.09 (−11.41, 17.60)	0.676	4.22 (−0.74, 9.19)	0.095
	Girls (overweight), *n* = 179	0.22 (−2.06, 2.50)	0.850	−3.43 (−16.321, 9.44)	0.601	−2.14 (−7.89, 3.62)	0.466
	Boys (obese), *n* = 47	−3.33 (−6.82, 0.36)	0.078	**−19.68 (−36.19, −3.16)**⁣^∗^	**0.020**⁣^∗^	−8.51 (−17.77, 0.76)	0.072
	Girls (obese), *n* = 47	−1.14 (−7.83, 5.55)	0.739	15.69 (−28.84, 60.20)	0.490	−3.76 (−19.17, 11.65)	0.632

*Note:* Bold at *p* value ≤ 0.05.

Abbreviations: BMI = body mass index, CI = confidence interval, IFA = iron–folic acid.

⁣^∗^Statistically significant.

**Table 6:** Impact of maternal supplement intake on childhood obesity markers: stratified by gender and gestational age.

Pregnancy supplement intake	Stratification	BMI		The sum of skinfold thickness		Waist circumference	
	Gender and gestational age	Coefficient (95% CI)	*p* value	Coefficient (95% CI)	*p* value	Coefficient (95% CI)	*p* value
Iron alone, yes	Boys (AGA), *n* = 944	0.07 (−0.47, 0.61)	0.796	−0.38 (−3.97, 3.20)	0.834	0.37 (−1.03, 1.77)	0.605
	Girls (AGA), *n* = 941	−0.33 (−0.83, 0.18)	0.203	−2.24 (−5.04, 0.56)	0.117	−1.12 (−2.32, 0.08)	0.067
	Boys (SGA), *n* = 143	0.66 (−0.88, 2.21)	0.402	1.59 (−8.06, 11.25)	0.746	1.08 (−2.72, 4.87)	0.578
	Girls (SGA), *n* = 141	**−1.67 (−3.22, −0.11)**⁣^∗^	**0.036**⁣^∗^	−7.59 (−21.12, 5.93)	0.271	−2.99 (−6.78, 0.80)	0.123
	Boys (LGA), *n* = 76	−0.86 (−2.48, 0.75)	0.294	−4.28 (−11.38, 2.82)	0.237	−2.98 (−7.26, 1.29)	0.172
	Girls (LGA), *n* = 97	0.17 (−1.12, 1.46)	0.795	−5.52 (−14.76, 3.72)	0.242	−0.63 (−4.67, 3.41)	0.760

Folic acid alone, yes	Boys (AGA), *n* = 944	0.14 (−0.56, 0.84)	0.696	−0.08 (−4.78, 4.62)	0.973	0.24 (−1.64, 2.11)	0.801
	Girls (AGA), *n* = 941	−0.65 (−1.31, 0.00)	0.051	−2.89 (−6.72, 0.92)	0.137	**−2.60 (−4.09, −1.10)**⁣^∗^	**0.001**⁣^∗^
	Boys (SGA), *n* = 143	1.03 (−1.51, 3.57)	0.428	1.29 (−12.84, 15.42)	0.858	1.18 (−4.71, 7.07)	0.694
	Girls (SGA), *n* = 141	**−3.11 (**−**4.46, −1.77)**⁣^∗^	**< 0.001**⁣^∗^	**−20.22 (**−**26.25,** −**14.19)**⁣^∗^	**< 0.001**⁣^∗^	**−8.28 (**−**11.50,** −**5.05)**⁣^∗^	**< 0.001**⁣^∗^
	Boys (LGA), *n* = 76	−0.08 (−3.21, 3.04)	0.959	−1.92 (−13.36, 9.52)	0.742	0.26 (−6.01, 6.53)	0.934
	Girls (LGA), *n* = 97	0.99 (−0.94, 2.92)	0.315	−2.88 (−15.29, 9.58)	0.653	0.96 (−4.48, 6.39)	0.730

IFA, yes	Boys (AGA), *n* = 944	0.35 (−0.41, 1.11)	0.362	1.50 (−3.75, 6.75)	0.575	−0.77 (−1.26, 2.818)	0.456
	Girls (AGA), *n* = 941	−0.48 (−1.21, 0.25)	0.199	−2.37 (−6.61, 1.87)	0.273	**−2.33 (**−**3.99,** −**0.68)**⁣^∗^	**0.006**⁣^∗^
	Boys (SGA), *n* = 143	1.03 (−1.51, 3.57)	0.428	1.29 (−12.84, 15.42)	0.858	1.18 (−4.71, 7.07)	0.694
	Girls (SGA), *n* = 141	**−3.47 (**−**4.76,** −**2.17)**⁣^∗^	**< 0.001**⁣^∗^	**−20.55 (**−**26.46,** −**14.64)**⁣^∗^	**< 0.001**⁣^∗^	**−8.31 (**−**11.63,** −**4.99)**⁣^∗^	**< 0.001**⁣^∗^
	Boys (LGA), *n* = 76	1.32 (−2.13, 4.76)	0.454	3.44 (−8.39, 15.28)	0.569	2.71 (−3.91, 9.33)	0.422
	Girls (LGA), *n* = 97	0.99 (−0.94, 2.92)	0.315	−4.06 (−17.50, 9.38)	0.554	−1.37 (−6.18, 3.43)	0.576

*Note:* Bold at *p* value ≤ 0.05.

Abbreviations: AGA = average for gestational age, BMI = body mass index, CI = confidence interval, IFA = iron–folic acid, LGA = large for gestational age and SGA = small for gestational age.

⁣^∗^Statistically significant.

## Data Availability

The data supporting this article's conclusions are not publicly available due to ethical restrictions. However, upon reasonable request, the corresponding author (M.G.) will make the data available.

## Supporting Information

Supporting Information 1 The authors provided a supporting figure (S1) presenting a DAG used for selecting controlling variables in the study. It includes key variables such as age, education, maternal health status and maternal lifestyle factors. The figure illustrates their relationships with the primary outcome and other covariates, helping to clarify the assumed causal pathways and inform the selection of appropriate controls in the analysis.

Supporting Information 2 A supporting table (S1) presents the results of sensitivity analyses using standardised BMI *z*-scores. Standardised BMI *z*-scores and raw BMI data did not reveal significant differences in the results.
