# Supplementary material for: Differential Impacts of Prenatal Supplement Intake on Childhood Obesity Markers, Stratified by Gender and Other Prenatal Factors
Source: J Obes. 2025 Feb 10;2025:3257488. doi: 10.1155/jobe/3257488 (PMC11832260; doi:10.1155/jobe/3257488)
Supplement: Supporting Information 1 — The authors provided a supporting figure (S1) presenting a DAG used for selecting controlling variables in the study. It includes key variables such as age, education, maternal health status and maternal lifestyle factors. The figure illustrates their relationships with the primary outcome and other covariates, helping to clarify the assumed causal pathways and inform the selection of appropriate controls in the analysis. [file 3257488.f1.docx]

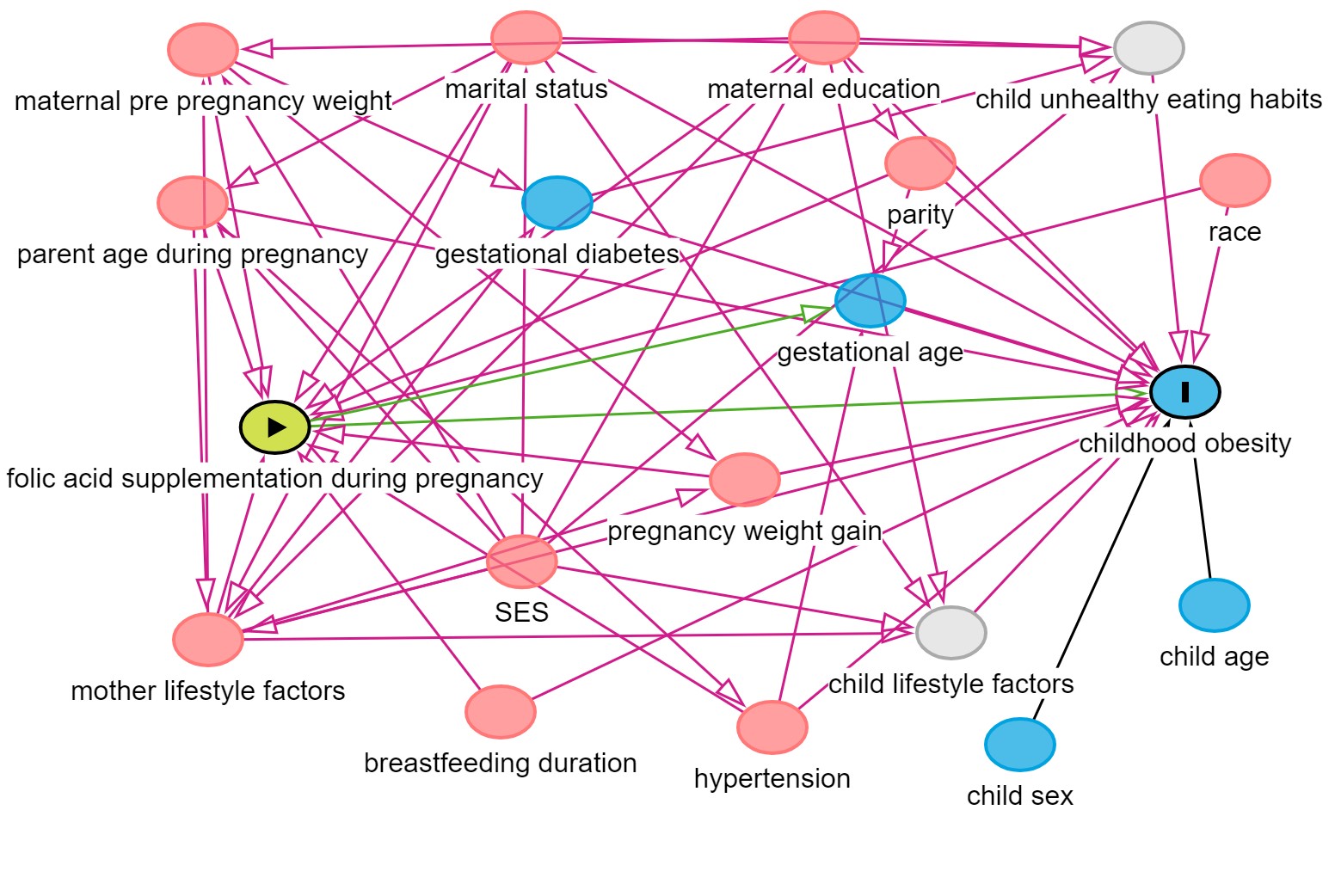


Figure S1: Direct acyclic graph (DAG) used to select controlling variables: SES, socio-economic status.
