# Supplementary material for: Differential Impacts of Prenatal Supplement Intake on Childhood Obesity Markers, Stratified by Gender and Other Prenatal Factors
Source: J Obes. 2025 Feb 10;2025:3257488. doi: 10.1155/jobe/3257488 (PMC11832260; doi:10.1155/jobe/3257488)
Supplement: Supporting Information 2 — A supporting table (S1) presents the results of sensitivity analyses using standardised BMI z-scores. Standardised BMI z-scores and raw BMI data did not reveal significant differences in the results. [file 3257488.f2.docx]

| Pregnancy supplement intake | Childhood outcomes, coefficient (95% CI), p-value | | |
| --- | --- | --- | --- |
|  | BMI Z-SCORES | | |
|  | All  n=2241 | Boys  n=1106 | Girls  n=1135 |
| Iron alone, Yes | -0.04 (-0.15, 0.06),  0.425 | -0.01 (-0.17, 0.14),  0.725 | -0.08 (-0.23, 0.06),  0.262 |
| Folic acid alone,  Yes | -0.07 (-0.22, 0.08),  0.360 | 0.08 (-0.14, 0.30),  0.462 | **-0.24 (-0.44, -0.04),**  **0.017*** |
| IFA, Yes | -0.03 (- 0.19, 0.13),  0.757 | 0.14 (-0.09, 0.37),  0.237 | **-0.23 (-0.45, -0.01),**  **0.042*** |

Table S1: Associations Between Pregnancy Supplement Intake and BMI z-Scores (Sensitivity Analysis).
